# A crustacean annotated transcriptome (CAT) database

**DOI:** 10.1186/s12864-019-6433-3

**Published:** 2020-01-09

**Authors:** Wenyan Nong, Zacary Y. H. Chai, Xiaosen Jiang, Jing Qin, Ka Yan Ma, King Ming Chan, Ting Fung Chan, Billy K. C. Chow, Hoi Shan Kwan, Chris K. C. Wong, Jian-Wen Qiu, Jerome H. L. Hui, Ka Hou Chu

**Affiliations:** 1 School of Life Sciences, Simon F.S. Li Marine Science Laboratory, State Key Laboratory of Agrobiotechnology, The Chinese University of Hong Kong, Hong Kong, China; 2 School of Life Sciences, Simon F.S. Li Marine Science Laboratory, The Chinese University of Hong Kong, Hong Kong, China; 3 School of Future Technology, University of Chinese Academy of Sciences, Hong Kong, China; 4 School of Pharmaceutical Sciences (Shenzhen), Sun Yat-sen University, Shenzhen, China; 5 School of Life Sciences, The Chinese University of Hong Kong, Hong Kong, China; 6 School of Life Sciences, State Key Laboratory of Agrobiotechnology, The Chinese University of Hong Kong, Hong Kong, China; 7 School of Biological Sciences, The University of Hong Kong, Hong Kong, China; 8 Department of Biology, Hong Kong Baptist University, Hong Kong, China

## Abstract

**Background:**

Decapods are an order of crustaceans which includes shrimps, crabs, lobsters and crayfish. They occur worldwide and are of great scientific interest as well as being of ecological and economic importance in fisheries and aquaculture. However, our knowledge of their biology mainly comes from the group which is most closely related to crustaceans – insects. Here we produce a de novo transcriptome database, crustacean annotated transcriptome (CAT) database, spanning multiple tissues and the life stages of seven crustaceans.

**Description:**

A total of 71 transcriptome assemblies from six decapod species and a stomatopod species, including the coral shrimp *Stenopus hispidus*, the cherry shrimp *Neocaridina davidi*, the redclaw crayfish *Cherax quadricarinatus*, the spiny lobster *Panulirus ornatus*, the red king crab *Paralithodes camtschaticus*, the coconut crab *Birgus latro*, and the zebra mantis shrimp *Lysiosquillina maculata*, were generated. Differential gene expression analyses within species were generated as a reference and included in a graphical user interface database at http://cat.sls.cuhk.edu.hk/. Users can carry out gene name searches and also access gene sequences based on a sequence query using the BLAST search function.

**Conclusions:**

The data generated and deposited in this database offers a valuable resource for the further study of these crustaceans, as well as being of use in aquaculture development.

## Background

**Fig. 1:**
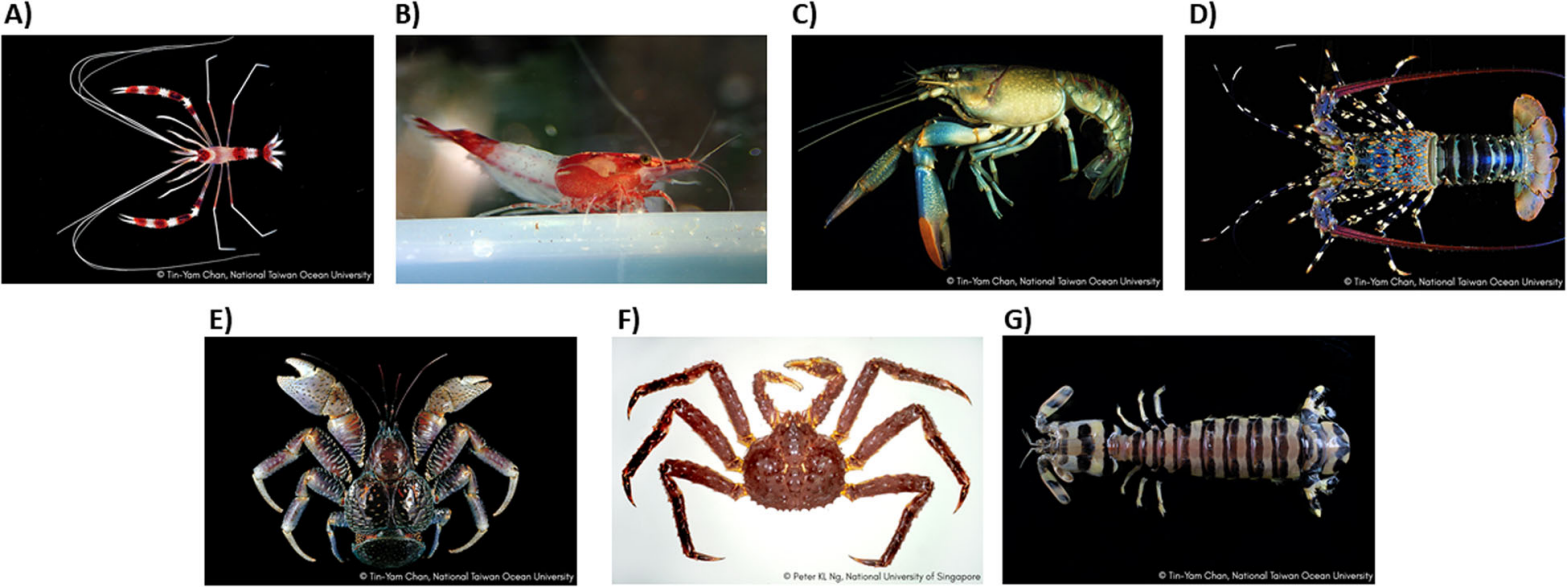
Crustaceans used in this study, including (**a**) coral shrimp *Stenopus hispidus*, (**b**) cherry shrimp *Neocaridina davidi*, (**c**) redclaw crayfish *Cherax quadricarinatus*, (**d**) spiny lobster *Panulirus ornatus*, (**e**) red king crab *Paralithodes camtschaticus*, (**f**) coconut crab *Birgus latro*, and (**g**) stomatopod zebra mantis shrimp *Lysiosquillina maculata*

The Arthropoda is a phylum containing the largest number (nearly 85%) of described living species in the world. For various historical reasons, most of our knowledge of their biology comes from insects, particularly fruit flies *Drosophila*. Crustacea (including shrimps, lobsters, crayfish, crabs) forms a large subphylum of arthropods now proven to be the closest relatives of Insecta. In the past decade, a substantial number of insect genomes have been sequenced across the different groups (e.g. beetle, wasp, bee, aphid, butterfly, and moth), especially in the course of the on-going 5000 insect genome project (i5k Consortium). By contrast, the genomic resources of crustaceans are relatively scarce, and are limited to a few species (e.g. [1–6]). Carcinology, or the study of crustaceans, benefits both basic science and the aquaculture industry, presently the fastest growing animal food-producing sector worldwide. Here, we generated a user-friendly database, the crustacean annotated transcriptome (CAT) database, which enables users to search for the annotated gene name as well as gene sequences based on sequence query. This database contains newly generated crustacean transcriptomic data from different developmental stages and the tissues of seven crustacean species, including a stomatopod mantis shrimp, two decapod shrimps, a crayfish, a lobster, and two anomuran crabs (Fig. 1).

## Construction and content

### Sample collection

Specimens of the seven crustacean species were acquired either from fish markets and aquarium shops in Hong Kong or from overseas sources (see details below). The creatures were then maintained in the laboratory before being dissected, as described below:

Coral shrimps (Decapoda: Stenopodidea: Stenopodidae: *Stenopus hispidus*) were sourced from an aquarium shop and maintained for over 2 weeks as mating pairs in separate 10-L seawater tanks at an ambient indoor temperature (20–26 °C) with diurnal lighting and environmental enrichments of moss and wood, and were fed with aquarist shrimp feed. Tissue samples were collected from a single adult female at the intermolt stage, while “whole body” samples were obtained from 50 to 100 early (no eye spot) and late (with eye spot) stage eggs obtained from two females separately.

Cherry shrimp (Decapoda: Caridea: Atyidae: *Neocaridina davidi*) were purchased from an aquarium shop in Hong Kong. Again, they were kept in 10-L freshwater tanks at an ambient indoor temperature with diurnal lighting, and fed with aquarist shrimp feed. Tissue samples were collected from a single female adult at the intermolt stage, while “whole-body” samples were obtained from a 15-day-old juvenile, as well as from ~ 20 early (no eye spot) and late (with eye spot) stage eggs (~ 6 eggs per replicate) from two females separately.

Red claw crayfish (Decapoda: Astacidea: Parastacidae: *Cherax quadricarinatus*) at different life history stages were sourced from a breeder in Queensland, Australia. The juvenile (~ 7–10 cm in length) and adult (15–18 cm in length) crayfish were acclimated for over 2 weeks in 100-L freshwater tanks at an ambient indoor temperature with diurnal lighting and enriched with hiding nets, and were fed aquarist shrimp feed. Tissue samples were collected from a single adult female at the intermolt stage, from a single juvenile, from 4 newborn larvae (less than 10 days old, 2 individuals per replicate) and from 6 early (orange) and 6 late (brown) stage egg (3 eggs per replicate).

Spiny lobsters (Decapoda: Achelata: Palinuridae: *Panulirus ornatus*) were purchased from a fish market in Hong Kong, and acclimated for 2 weeks in 500-L tanks in an outdoor enclosure at 25–30 °C and fed with live clams. Tissue samples were collected from a single adult female at the intermolt stage.

Adult male coconut crabs (Decapoda: Anomura: Coenobitidae: *Birgus latro*) were purchased and imported from a fish market in Okinawa, Japan.. The crabs were fed a diet of coconut meat and boiled root vegetables while acclimating for 2 weeks in a controlled environment in a large outdoor enclosure at 25–30 °C. The enclosure was enriched with damp straw, reptile lights on diurnal control and a pool of running fresh water, and a humidifier maintained a relative humidity of 70–80%. Tissue samples were collected from a single individual.

Adult male king crabs (Decapoda: Anomura: Lithodidae: *Paralithodes camtschaticus*) were imported from Alaska and fed with live clams while acclimating for 2 weeks in 100-L seawater tanks kept at 4 °C in a dark room. Tissue samples were collected from a single individual.

Zebra mantis shrimp (Stomapoda: Lysiosquillidae: *Lysiosquillina maculata*) were purchased from a fish market in Hong Kong and acclimated for 2 weeks in 100-L seawater tanks at ambient indoor temperature with diurnal lighting and 20 cm of sand, and were fed with live fish. Tissue samples were collected from a single adult female at the intermolt stage.

Tissue samples of gill, eye stalk, ovary (female only), hepatopancreas, and muscle were obtained from adults of all target species and juveniles of crayfish. Gill tissues were dissected, pooled and homogenised. Tissue from eyestalks were dissected, avoiding the pigmented retina and discarding the exoskeleton. Ovary tissues were collected from mature females. Hepatopancreas tissues were taken at distant tubules from the midgut caecae to avoid heavy bacterial contamination. Muscle was isolated from the abdomen from all the shrimp and crayfish species (including stomatopod) and from the large chela of crabs. Duplicate biological samples were collected. Tissue samples from adult and the “whole body” of juvenile animals, larvae, and eggs were frozen in liquid nitrogen and then stored at − 80 °C before total RNA extraction.

### RNA extraction and sequencing

Total RNAs were isolated using the miRVana microRNA Isolation Kit (Thermo Fisher Scientific). RNA concentration and quality were assessed by a NanoDrop Flourospectrometer (Thermo Scientific). At least 5 μg of total RNA for each sample were enriched by ribo-reduction using Ribo-Zero rRNA removal kits (Epicentre). Transcriptome libraries were created using TruSeq Stranded RNA Library Prep Kit v2 (Illumina) by Theragen Bio Institute in Korea, followed by 150 bp paired-end sequencing on an Illumina HiSeq 4000 platform to obtain at least 51 million clean reads (after filtering and trimming).

### Transcriptome assembly and annotation

Raw sequencing reads from 71 transcriptomes were pre-processed with quality trimmed by trimmomatic (v0.33 with parameters “ILLUMINACLIP:TruSeq3-PE.fa:2:30:10 SLIDINGWINDOW:4:5 LEADING:5 TRAILING:5 MINLEN:25”, [7]), followed by de novo transcriptome assembly using Trinity (v2.4.0, [8, 9]) with the options “--SS_lib_type RF --normalize_reads” and other default parameters. All biological duplicates were combined to carry out the de novo assembly and estimation of transcript abundance using the script “align_and_estimate_abundance.pl” of the Trinity software with “--est_method RSEM --aln_method bowtie” (v1.1.2, [10]). Coding regions within transcripts were annotated using TransDecoder (v5.0.2 [11];), and functional annotation and analyses were carried out using Trinotate (v3.1.1, [12]). A summary of the assembled transcriptomes is shown in Table 1.

**Table 1:**
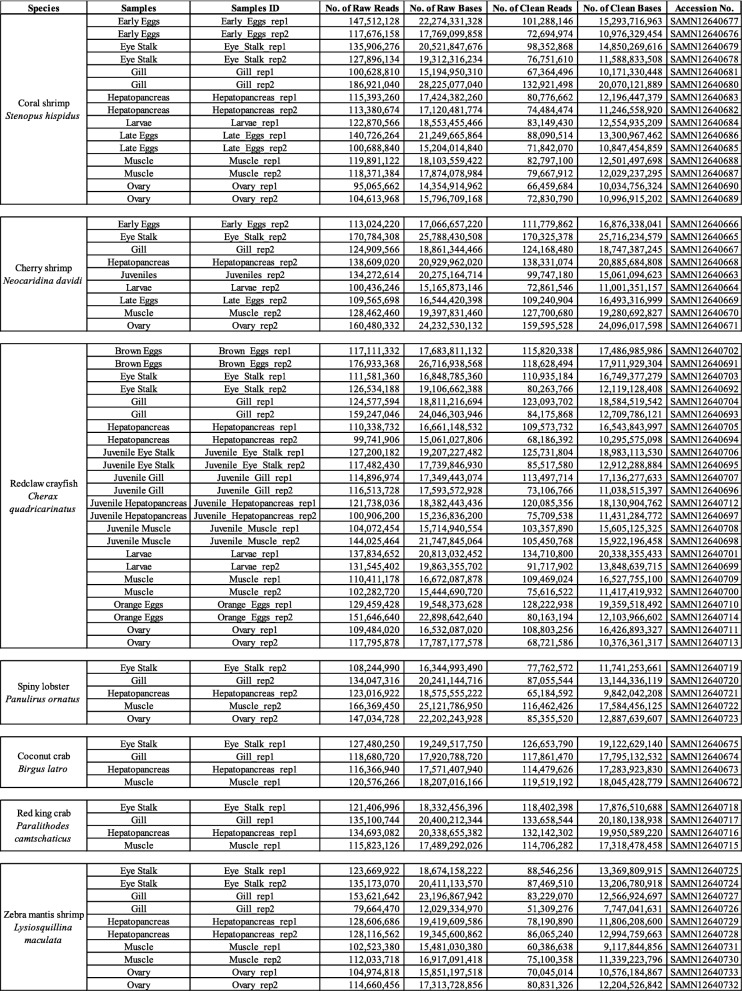
Transcriptomes generated in this study

### Utility and discussion

#### Website construction

The Crustacean Annotated Transcriptome (CAT) database is available at http://cat.sls.cuhk.edu.hk/. It was built using CodeIgniter Web Framework. CodeIgniter (https://www.codeigniter.com/) is a powerful PHP framework with a tiny footprint. The website provides researchers with several tools for transcriptome visualization, gene search, and gene blast.

##### Transcriptome visualisation

Gene expression data of various samples in each species can be visualised through the Degust (https://github.com/Victorian-Bioinformatics-Consortium/degust) toolset [13]. It allows the comparison of gene expression between different tissues of the same species. The users can browse differentially expressed genes (DEGs) between samples within the same species, perform their own DEG analysis, or analyse expression profiles using the inbuilt server.

##### Gene sequence search

The database contains 462,877 pieces of gene annotation information (coral shrimp: 57240, cherry shrimp: 92956, red claw crayfish: 99100, spiny lobster: 28805, coconut crab: 72729, red king crab: 73144, zebra mantis shrimp: 38903). The users can search a gene of a certain species by querying the “gene id” or “gene name” and selecting the species in the gene search section. After users submit their request, the results will be displayed in a table. The number of results will be shown at the top of the table. The table will list the general information of all matched genes, including the gene id, gene name and species information. Clicking on the “gene id” or “gene name” will bring users to a detailed information page of the gene. The nucleic acid sequence derived from de novo assembly, protein sequence deduced from assembled transcripts, and the expression of the gene in each sample can be viewed on the page.

##### Gene blast

The user can input or upload query sequence(s) in fasta format, select the corresponding species database and the blast type to perform the gene blast. Hits will be listed in the result table. The users can browse the detailed information of the hit genes by clicking on the hit IDs.

## Conclusions

Carcinology benefits both the basic science and the aquaculture industry. We have here generated a platform (CAT) in hosting 71 new transcriptomes generated for seven species of decapod crustaceans and a stomatopod. CAT is constructed in a way aiming to facilitate research on this important branch of life, and will continue to be updated, to host more crustacean genomic resources in the future.

## Data Availability

The transcriptome data were deposited in NCBI under BioProjects PRJNA562428.

## Abbreviations

BLAST: Basic Local Alignment Search Tool
CAT: Crustacean annotated transcriptome database
DEGs: Differentially expressed genes
IDs: Identifications
RNA: Ribonucleic acid
rRNA: Ribosomal ribonucleic acid
